# Metachromatic Leukodystrophy: Diagnosis, Modeling, and Treatment Approaches

**DOI:** 10.3389/fmed.2020.576221

**Published:** 2020-10-20

**Authors:** Alisa A. Shaimardanova, Daria S. Chulpanova, Valeriya V. Solovyeva, Aysilu I. Mullagulova, Kristina V. Kitaeva, Cinzia Allegrucci, Albert A. Rizvanov

**Affiliations:** ^1^Institute of Fundamental Medicine and Biology, Kazan Federal University, Kazan, Russia; ^2^Shemyakin-Ovchinnikov Institute of Bioorganic Chemistry, The Russian Academy of Sciences, Moscow, Russia; ^3^School of Veterinary Medicine and Science (SVMS) and Biodiscovery Institute, University of Nottingham, Nottingham, United Kingdom

**Keywords:** lysosomal storage diseases, metachromatic leukodystrophy, arylsulfatase A, sulfatide, replacement therapy, gene therapy, bone marrow transplantation, mesenchymal stem cells

## Abstract

Metachromatic leukodystrophy is a lysosomal storage disease, which is characterized by damage of the myelin sheath that covers most of nerve fibers of the central and peripheral nervous systems. The disease occurs due to a deficiency of the lysosomal enzyme arylsulfatase A (ARSA) or its sphingolipid activator protein B (SapB) and it clinically manifests as progressive motor and cognitive deficiency. ARSA and SapB protein deficiency are caused by mutations in the ARSA and PSAP genes, respectively. The severity of clinical course in metachromatic leukodystrophy is determined by the residual ARSA activity, depending on the type of mutation. Currently, there is no effective treatment for this disease. Clinical cases of bone marrow or cord blood transplantation have been reported, however the therapeutic effectiveness of these methods remains insufficient to prevent aggravation of neurological disorders. Encouraging results have been obtained using gene therapy for delivering the wild-type ARSA gene using vectors based on various serotypes of adeno-associated viruses, as well as using mesenchymal stem cells and combined gene-cell therapy. This review discusses therapeutic strategies for the treatment of metachromatic leukodystrophy, as well as diagnostic methods and modeling of this pathology in animals to evaluate the effectiveness of new therapies.

## Introduction

Metachromatic leukodystrophy (MLD) is an autosomal recessive hereditary neurodegenerative disease belonging to the group of lysosomal storage diseases (LSDs). MLD is one of the most common leukodystrophies, and has a prevalence rate of 1 in 40,000–160,000 worldwide. In some isolated populations, the incidence of MLD is much higher. For example, in the group of Habbanite (Jews) it is estimated at 1 in 75, among the Navajo Indian people at 1 in 2,500, and among the Arab groups of Israel it is estimated at 1 in 8,000 (1).

The disease is characterized by the damage of the myelin sheath that covers most of the nerve fibers of the central (CNS) and peripheral nervous systems (PNS), resulting in progressive motor and cognitive impairment as clinical manifestations (2).

With LSD, uncleaved macromolecules accumulate within lysosomes and spread out in the other cellular compartments over time. The disease progression and lysosome dysfunction lead to the secondary deficiency of other enzymes, resulting in a complete disruption of the lysosomal system functioning and the death of the affected cells (3, 4).

MLD takes its name from the presence of metachromatic granules in the affected cells, formed as a result of the accumulation of sulfatides and sphingolipids presented in myelin. With MLD, sulfatides accumulate in oligodendrocytes, microglia, some CNS neurons, Schwann cells, PNS macrophages. They also accumulate in the cells of internal organs, such as the gall bladder, thus increasing the risk for malignant neoplasms of this organ (5–12). Morphological changes in the endoplasmic reticulum (EPR) and mitochondria in Schwann cells are also described (13).

MLD causes demyelination to occur, leading to impaired motor function, spastic tetraparesis, ataxia, spasms, optic atrophy, and cognitive impairment (14, 15). However, the exact mechanisms of demyelination in MLD remain unknown. Possible causes are an increase in the sulfatides and a decrease in its cleavage products, which lead to instability of the myelin sheath (16). In addition, sulfatides cause accumulation of calcium in the cell cytoplasm, which results in a change in calcium homeostasis, cell stress, and apoptosis (10).

The accumulation of sulfatides leads to neuronal degeneration, astrocyte dysfunction and may trigger an inflammatory response. Several studies support the role of inflammation in MLD. For instance, increased levels of monocyte chemoattractant protein 1 (MCP-1), interleukin (IL)-1 receptor antagonist (IL-1Ra), IL-8, macrophage inflammatory protein 1β (MIP-1β) and vascular endothelial growth factor (VEGF) are detected in both plasma and cerebrospinal fluid (CSF) of patients with MLD. The described cytokines can function as biomarkers to detect MLD at early stages and to analyze disease progression (17). With MLD, there may be a lack of correlation between demyelination and the presence of metachromatic material. Thus, complement activation via an alternative pathway enhances myelin damage, thus inducing or enhancing the immune response against myelin (18). In the PNS the sulfatide accumulation and demyelination can induce the release of inflammatory cytokines, activate endoneurial macrophages and also recruit inflammatory myeloid cells and lymphocytes from the periphery. These processes are involved in apoptosis and can stimulate the progression of demyelination and neuroinflammation, which are also observed in some other metabolic neurodegenerative diseases (13). Microglia is also assumed to play an important role in the development of MLD. It has been shown that the microglia immune phenotype changes at the early stages of MLD development and this precedes oligodendrocyte degeneration and myelin damage (19).

## Cause of MLD: ARSA Enzyme and Sphingolipid Activator Protein B

MLD is caused by the deficiency of arylsulfatase A lysosomal enzyme (ARSA) (OMIM 250100) and sphingolipid activator protein B (SapB or saposin B) (OMIM 249900) as a consequence of mutations in the *ARSA* and *PSAP* genes, respectively. About 261 unique mutations in the *ARSA* gene (https://databases.lovd.nl/shared/genes/ARSA) and 64 unique mutations in the *PSAP* gene, (https://databases.lovd.nl/shared/genes/PSAP) leading to the development of MLD, are reported to date (20). ARSA deficiency cannot be compensated by other enzymes (21). The *ARSA* gene (Gene/Locus MIM 607574) is located on chromosome 22q13.33 and consists of 9 exons. ARSA is synthesized as a pre-protein, then the signal peptide is cleaved in the EPR, resulting in the formation of a mature ARSA protein consisting of 489 amino acids with a molecular weight of 51,908 Da (22).

For all lysosomal enzymes, including ARSA, lysosome targeting is ensured by the presence of mannose 6-phosphate (M6P) residues, which are added to the lysosomal enzymes as it passes through the Golgi complex (GC). After entering the lysosome, the M6P is cleaved off the enzyme (23, 24). At lysosomal acidic pH, ARSA exists as a homo-octamer composed of 4 dimers arranged in a ring-like structure. However, it is predominantly dimeric at neutral pH. The physiological substrate of ARSA is sulfatide (3-O-sulfo-galactosylceramide, cerebroside-3-sulfate, galactosyl-3-sulfate ceramide) (22).

Sulfatides are one of the most common sphingolipids in myelin. Sulfatides are present in myelinating cells (oligodendrocytes and Schwann cells). They perform important functions in the differentiation of oligodendrocytes and are involved in the formation of paranodal regions (25), in signaling and maintenance of myelin's structure and function (26). The synthesis of sulfatides begins in the EPR, where galactose is transferred to ceramide from uridine diphosphate galactose. Then, the resulting galactosylceramide (GalC) is transported to the GC, where sulfatide is finally formed after transfer of sulfate from 3′-phosphoadenosine-5′-phosphosulfate (PAPS) to GalC by galactose-3-O-sulfotransferase-1 (Gal3st1).

Sulfatides vary in the acyl chain length and in the extent of hydroxylation. The ratio of different sulfatides varies with the development of the nervous system, and also depends on the cell type (27). For example, short chain fatty acid sulfatides (e.g., C16:0 and C18:0) are more common in neurons and astrocytes, whereas long chain fatty acid sulfatides (e.g., C24:1 and C24:0) are present in myelin (28).

**Figure 1:**
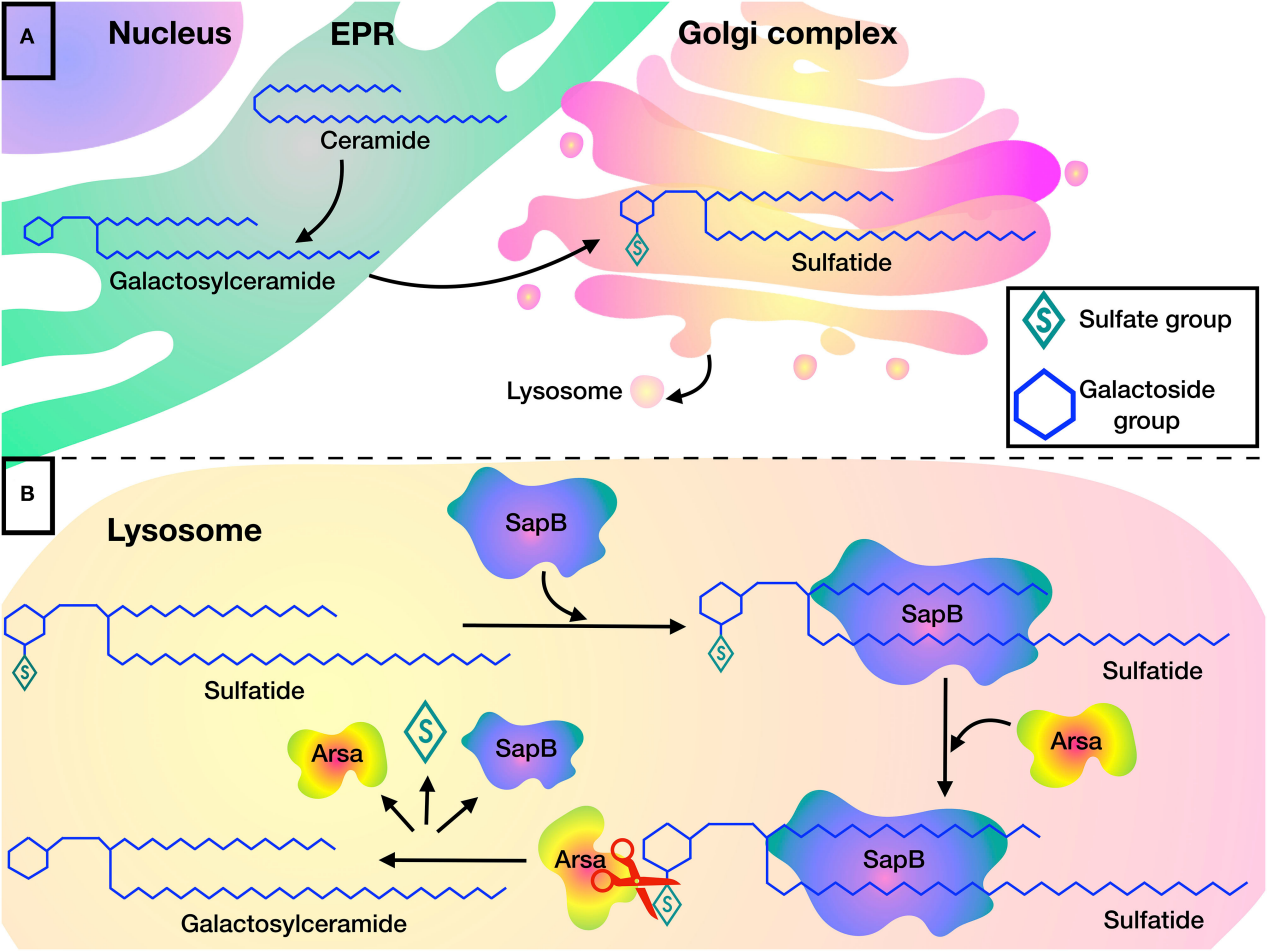
Sulfatide metabolism: **(A)** Biosynthesis of sulfatides begins in the EPR, where galactosylceramide is formed by transferring galactose to ceramide, after which galactosylceramide is transported to the Golgi complex where sulfatides are formed by addition of a sulfate group. **(B)** Degradation of sulfatides occurs in the lysosome. SapB is required for the presentation of sulfatide to the active site of ARSA. ARSA hydrolyzes sulfatide to galactosylceramide by cleaving the sulfate group.

Sulfatides are cleaved in the lysosome, where ARSA hydrolyzes the sulfate group. The cleavage reaction can be carried out only if the sulfatide forms a complex with SapB, which makes the hydrophobic substrate soluble (Figure 1) (29). Indeed, detergents such as sodium taurodeoxycholate, can be used instead of an activator protein in *in vitro* experiments (22, 30, 31).

ARSA is a homo-octamer composed of a tetramer of dimers, which belongs to the family of sulfatases that carry C (alpha)-formylglycine, formed as a result of post-translational modification by oxidation of conserved cysteine or serine residues. Sulfatases are widespread hydrolases that hydrolyze the sulfate ester bonds of various compounds and it is believed that the presence of C (alpha)-formylglycine is necessary for sulfatase activity (32). The crystal structure of ARSA and the proposed hydrolysis mechanism were reported by the study of Saenger et al. in 1998 (22), demonstrating that ARSA-mediated hydrolysis of substrates is a multi-step mechanism involving nucleophile activation, nucleophilic attack, and S-O bond cleavage (33).

ARSA cleaves a wide range of substrates, including synthetic ether sulfates with an optimal pH of 5–6 (22). Artificial substrates are used to evaluate enzyme activity in research and diagnostics. For example, ARSA hydrolyzes an artificial chromogenic substrate ρ.nitrocatechol-sulfate (ρ.NCS) (34).

Just 10-15% of the ARSA enzymatic activity is sufficient for sulfatide degradation and maintenance of normal life (35, 36). Indeed, ARSA pseudodeficiency characterized by a decrease of ARSA enzymatic activity up to 5–20% does not result in a diseased phenotype as the residual enzymatic activity is sufficient for substrate catabolism (37). In some cases, a shortened protein can be synthesized, but without changing the activity and stability (38). Presumably, ARSA pseudodeficiency may be associated with cardiovascular problems, renal failure and type 2 diabetes, however these associations need to be confirmed (39).

The *PSAP* (Gene/Locus MIM 176801) gene is located on chromosome 10q21.1 instead, and consists of 15 exons (20). The *PSAP* gene encodes a precursor protein, prosaposin (pSap), consisting of 524 amino acids. As a result of the cleavage of the precursor protein, four types of saposin are formed: SapA, SapB, SapC, and SapD. These saposins are sphingolipid activator proteins and are necessary for the functioning of some lysosomal enzymes (40).

Mutations in the *PSAP* gene can cause either a single saposin deficiency or a deficiency of the whole pSap protein, which result in severe lethal neurodystrophy (OMIM 611721). SapB deficiency leads to the development of MLD. Defects in SapA and SapC lead to atypical forms of Krabbe disease (OMIM 611722) (41) and Gaucher disease (OMIM 610539) (42), respectively. SapD deficiency is not described in clinical practice; however, SapD^−/−^ mice defective in SapD were obtained (43, 44). The phenotype of SapB defect is similar to classical MLD phenotype caused by ARSA deficiency. In addition, SapB is required for the cleavage of globotriosylceramide digalactosylceramide and GM3-ganglioside substrates by other specific lysosomal enzymes (40).

## MLD Classification

MLD is classified into late infantile, juvenile, and adult types (17, 45). The clinical manifestations and a degree of neurodegeneration in MLD are diverse and depend on the type of mutation and the level of enzyme deficiency (16). It is hypothesized that the lower the enzyme activity the earlier the disease manifests itself, however such association has yet to be fully demonstrated. Two types of alleles that cause the development of MLD can be distinguished: null alleles, which encode an inactive enzyme, and R-alleles, which encode an enzyme with residual activity. To date, the following correlation of the genotype and phenotype is currently accepted: genotypes containing two 0-alleles cause an infantile form; genotypes in which null and R alleles are combined cause juvenile form; genotypes containing two R-alleles cause the adult form of MLD (36, 46, 47).

Nevertheless, so far it has been difficult to determine the exact relationship of the genotype with the residual activity of the ARSA enzyme and phenotype. It is important to note that the onset of the disease and phenotype may differ even in siblings with the same mutation. The reasons for this finding remains unknown. It is assumed that other biochemical and epigenetic factors may influence the clinical phenotype of the disease (48).

The most common form is the late infantile, which is found in 50–60% of all patients. On the other hand, 20–30% of patients are diagnosed with the juvenile form and the adult form is the most rare, affecting 15–20% of patients with MLD (49).

Clinical manifestation of late infantile MLD begins up to 30 months of age. This form of MLD is considered the most severe, characterized by lack of or minimal residual ARSA activity, which entails rapid neurodegeneration. The disease usually starts with either gait abnormalities or delay in early motor milestones. The initial stages can be characterized by muscle weakness, pathological movements, neuropathy, as well as development regression. Peripheral neuropathy is observed, which is associated with a decrease in motor and sensory nerve conduction (14). Subsequently, peripheral neuropathy, regression of speech, cognitive, motor abilities, deterioration of fine motor skills, spasticity, ataxia, convulsions, visual, and hearing impairment often occur. The terminal stage is characterized by the development of severe psychomotor retardation and often there is an atrophy of the optic nerve, pseudobulbar and bulbar palsy and, at least, swallowing and breathing alteration. The death of patients with late infantile form occurs in childhood (50, 51).

In patients with a late infantile MLD form CNS symptoms are often preceded by rapidly progressive peripheral neuropathy, which is characterized by clumsiness, muscle weakness, sensory deficits and areflexia. There is also a significant slowdown in motor and sensory conduction. However, as the disease progresses, the symptoms of peripheral neuropathy are gradually masked by the development of spastic tetraparesis and other manifestations of CNS dysfunction. Other PNS symptoms include neurogenic bladder dysfunction, neuropathic pain, and severe foot deformities (13).

The juvenile form develops between the ages of 3 and 16 and is characterized by a less pronounced clinical manifestation in comparison with the late infantile form. In the juvenile form, cognitive impairment and behavioral changes are often observed, followed by deterioration of central and peripheral motility and epilepsy. The disease manifestation begins with the behavioral problems, psychiatric symptoms, delay in fine motor skills and impaired attention concentration. Problems with a child's ability to learn are also often observed. As the disease develops, problems with motor function arise, muscle hypertonia, spastic posturing are often observed (45, 52, 53). In late infantile and early juvenile forms, a rapid disease progression is observed and in the absence of therapy death occurs within a few years since the disease onset (15, 17). However, with supportive treatment, including a gastric tube insertion for feeding and antibiotic therapy during infections, patients could survive in a vegetative state for years (14, 54).

Clinical manifestation of the adult form of MLD begins in late adolescence, usually after 16 years of age. Adult MLD is the less severe form of the disease. In the adult form, psychoses (55), cognitive and behavioral impairment (53, 56), ataxia, polyneuropathy, and epileptic seizures (57) are found. Patients experience depressive disorder and sudden mood swings (58). Another typical feature is psychotic symptoms, such as hallucinations and illusions, which may be associated with a violation of cortico-cortical and cortico-subcortical connections, especially involving the frontal lobes (5). Adult MLD is the least common form of MLD, and it is often mistakenly diagnosed as early-onset dementia (58) or schizophrenia (56, 59) because of its slow progression. Slow disease progression with periods of relative stability and regression is typical of an adult MLD. The final stage of the disease is similar to the late infantile and juvenile forms. Patient's life expectancy is about 20–30 years after diagnosis (1).

## MLD Diagnosis

In most cases, MLD is not included in the fetus and newborn genetic screening tests given that MLD is a rare disease. Commonly, the disease is diagnosed after birth, depending on the form of MLD. Only in cases where the parents know that their family carries the mutation (family history, development of the disease in previous children), diagnosis and treatment can begin. However, early diagnosis is critically important. The introduction of prenatal diagnosis and newborn screening could increase therapy efficacy, since the effectiveness of the treatment is significantly reduced after the onset of symptoms (60).

Prenatal diagnostics can be performed by measuring the activity of ARSA in amniotic fluid cells and evaluating the accumulation of sulfatides to exclude ARSA pseudo-deficiency (61). Reliable and accurate results are also obtained by determining the activity of the enzyme in chorionic villus samples (62, 63). In addition, the determination of genetic mutations in the fetus is an easy diagnostic method to eliminate pseudo-deficiency (64–66). However, the determination of enzyme activity and the assessment of sulfatide accumulation are more informative methods as mechanisms of correlation between genotype and phenotype are still not clearly understood. A recent pilot screening has been carried out by measuring ARSA activity in dried blood spots or in white blood cells using natural substrates (d3-C18: 0-sulfatide) (31). A protocol for the screening is currently under large-scale investigation so that newborn screening for MLD may become routine practice in the near future.

Diagnosis of late onset forms of MLD is often more difficult. For instance, proper diagnosis is problematic as adult MLD is often mistaken for schizophrenia (56) or other types of mental disorders (53, 58, 67), therefore delaying the beginning of therapy.

To date, MLD is diagnosed by clinical manifestations, using genetic analysis for mutations in the *ARSA* and *PSAP* genes (68), magnetic resonance imaging (MRI) of the brain (69–71) and biochemical tests of the ARSA enzymatic activity in skin fibroblasts, leukocytes and urine of patients (31, 72). Moreover, approaches such as determining the level of sulfatides in plasma and urine by mass spectrometry (73), quantitative determination of metabolites in tissues by magnetic resonance spectroscopy (MRS) (74) and the evaluation of the peripheral nerve size using ultrasound (75) can provide additional information, both for the diagnosis and the evaluation of MLD treatment effectiveness.

Detection of mutations by sequencing is the most accurate method for diagnosing hereditary genetic diseases. However, diagnosis can be made using other methods (e.g., determination of ARSA activity or sulfatide levels as well as MRI) since not all the mutations associated with the disease have been currently identified (65, 66, 68, 76).

The next reliable method to monitor the disease course and the treatment effectiveness is to determine the ARSA enzyme activity. For this, there are special substrates that allow you to measure the level of ARSA activity: 4-methylumbelliferyl sulfate (4-MUS) and ρ.NCS are synthetic substrates for ARSA and arylsulfatase B (ARSB). ARSB, like ARSA, is a lysosomal enzyme encoded by the *ARSB* gene. Mutations in the *ARSB* gene lead to the accumulation of glycosaminoglycans and cause type VI mucopolysaccharidosis (77). A 4-MUS fluorescent substrate is preferably used to determine ARSB activity. To determine ARSA activity using 4-MUS, AgNO_3_ is added to the reaction mix to inhibit ARSB activity. However, inhibition is incomplete, therefore, such an analysis is not suitable for low ARSA activity quantification (78).

ARSA activity can also be determined with a spectrophotometric method using the ρ.NCS substrate, which is hydrolyzed by both ARSA and ARSB. To determine only the ARSA activity, ARSB are inhibited either by adding 0.25 mM sodium pyrophosphate (79), or by incubating the reaction mixture at 0°C, providing almost complete ARSB inhibition (<1% of ARSB activity remains) and a high selectivity for ARSA. Thus, ρ.NCS is a more preferred synthetic substrate for determining ARSA activity. According to previously described protocols (72), ARSA activity can be reliably determined in fibroblasts, leukocytes of patients' blood and urine (78).

Natural substrates (d3-C18: 0-sulfatide) can also be used to measure ARSA activity. In this case, the analysis is carried out using liquid chromatography and tandem mass spectrometry, since it is impossible to determine the activity by the colorimetric and fluorometric methods used in the case of artificial substrates. The advantage of natural substrates over artificial substrates is specificity to ARSA (31, 80).

Magnetic resonance imaging is a very sensitive and highly informative method that allows early identification of white matter damage and evaluation of the disease (69, 81). Late infantile MLD is characterized by abnormalities of nerve conduction and demyelinating neuropathy, as well as late brain demyelination in MRI (14, 82). In the juvenile form, the central and periventicular white matter are firstly affected. As the disease progresses, subcortical structures of white matter may also be affected. In extremely severe cases, projection fiber damage as well as the appearance of a “tigroid pattern” (bands that are associated with the preservation of myelin in the perivenular areas) are observed. The corpus callosum may also be affected (69–71). Changes in the cerebellum and myelination of subcortical U-fibers are usually not observed. If U-fibers are affected, severe motor dysfunction will develop (82).

With MLD, the amount of sulfatides in the biological fluids of patients is increased, thus making possible to determine the level of sulfatides in plasma and urine using mass spectrometry. It has been shown that in patients with MLD the level of all 14 plasma sulfatide isoforms is elevated. The level of C18 sulfatide showed the most significant difference in the plasma of patients with MLD Comparison of the total urine and plasma sulfatides in patients with MLD showed that urine sulfatide level is significantly higher (73). The results prove that this method minimizes the false-positive result in ARSA pseudodeficiency (27). Therefore, this method could effectively be used in the addition to measuring enzyme activity and determining genetic mutations in patients for the most reliable and informative result.

Biochemical composition changes in the tissue can be determined using MRS by quantifying the metabolites, including changes in the composition of compounds in the white matter. Patients with MLD have decreased levels of N-acetyl aspartate (NAA) and glutamate (Glu), and increased levels of myo-inositol (Ins) and lactate (Lac). According to the literature, a decrease in the levels of NAA and Glu indicates neuroaxonal damage, whereas an increase in Lac may indicate energy deficiency, macrophage/microglia activation. In contrast, it is believed that Ins can have a positive effect on the functioning of the nervous system, however this remains to be investigated. Thus, the method allows determining abnormal concentrations of chemical compounds in a white matter, which can be used to diagnose and predict the clinical course of MLD (74). Although this method is not commonly accepted for the MLD diagnosis, it can be useful to investigate pathological changes for research purposes.

With MLD, an increase in size of peripheral nerves occurs, which can be detected by ultrasound. However, an increase in peripheral nerves is also observed with many metabolic disorders, inflammatory processes and other pathologies. Therefore, ultrasound does not allow a definitive diagnosis of MLD (75).

Nerve conduction studies (NCS) are also an important method for MLD diagnosis, which allow assessing the peripheral nervous system of patients, in particular the speed of nerve conduction and the amplitude of nerve action potential (83). Since the first signs of the late infantile form of MLD are nerve conduction disorders and demyelinating neuropathy, this method is necessary both for the initial diagnosis and for evaluating treatment efficiency (84).

## MLD Modeling

As MDL is characterized by different clinical manifestations, effective modeling of the disease is important to study the genetic basis of the disease, disease progression, diagnosis, and therapy.

Genetically modified mice have been used to model the disease, as no animal model with naturally occurring MDL has been described. The first MLD genetically modified mouse was obtained by Hess et al. in 1996 by homologous genetic recombination, affecting the expression of ARSA (a neomycin cassette was inserted into exon 4 of the *ARSA* gene, which shifted the open reading frame). ARSA-deficient mice show all signs of the disease (sulfatide accumulation in neuronal and non-neuronal tissues, axon size reduction, astrogliosis, loss of acoustic ganglion neurons, and altered morphology of Purkinje cells), but had a normal life span. In addition, the resulting mice had no significant myelin disorders, white matter defects, and peripheral neuropathy (85). Since this mouse model has impaired expression of the functional ARSA enzyme, it is considered to be equivalent to the late infantile MLD and has been used for the past two decades to study the effectiveness of new MLD treatments (2). A limitation of the described MLD mouse model is the insufficient demyelination in both the CNS and the PNS. Therefore, transgenic ARSA^−/−^ mice with overexpression of the sulfate-synthesizing enzyme Gal3st1 in myelinating cells have been created. Overexpression of Gal3st1 in oligodendrocytes and Schwann cells of ARSA^−/−^ mice leads to a significant increase in the accumulation of sulfatides in the nervous system and to the development of myelin pathology in the CNS and especially in the PNS (86). The study of the therapeutic efficacy of new drugs begins at the age of 4, 8, or 12 months of ARSA^−/−^ mice. These ages are suitable because they resemble pre-symptomatic, early clinical and late stages of the disease, characterized by the absence of detectable neuronal dysfunction (4th month), the first signs of behavioral changes and impaired neural conductivity (8th month) and more severe neurological symptoms (12th month) (87).

SapB (B^−/−^) deficient mouse models have also been produced by a knock-in of cysteine 4 to create a phenylalanine substitution in exon 7 of the prosaposin locus that corresponds to the saposin B domain. Progressive accumulation of glycosphingolipids, mainly sulfatides, in the CNS and PNS has been described in B^−/−^ mice. In the brain and spinal cord, sulfatides were detected in microglial cells and oligodendrocytes (88). A mouse model with a combined deficiency of SapA and SapB (AB^−/−^) has also been created. AB^−/−^ mice have been obtained using point mutations by analogy with the B^−/−^ mouse strain and characterized by impaired motor function, abnormal motor activity and tremor and have had a shorter lifespan (~96 days) compared to B^−/−^ mice (~644 days) (89).

More recently, more close-to-patients models have been developed using the induced pluripotent stem cells (iPSCs) technology. Reprogramming of fibroblasts isolated from the skin of MLD patients into iPSC has been proposed for the *in vitro* modeling of MLD and for evaluating the effectiveness of ARSA activity restoration in affected cells. Further differentiation of iPSCs into a mixed population of neuroepithelial progenitor cells, neurons, astrocytes and oligodendrocytes has allowed the recapitulation of pathological processes occurring in ARSA deficient cells at the morphological, molecular and biochemical levels (90). Therefore, this new approach opens new avenues for the study of MDL using a clinical relevant model of the disease.

## MLD Therapy

There are no approved therapies for MLD to date. The main difficulty in the treatment of diseases affecting the nervous system arises from the poor permeability of the BBB (blood-brain barrier), which restricts access of therapeutic compounds during systemic administration and results in low effectiveness of many therapeutic approaches (91, 92). It is important to note that to prevent MLD progression, it is necessary to ensure the distribution of the drug throughout the nervous system, including the PNS.

The problem of overcoming the BBB could be solved, for example, by direct injection of the recombinant ARSA enzyme or viral vectors encoding the wild-type gene of the missing enzyme into the brain. The virus-based approach has often led to long-term and stable transgene expression, as confirmed by a number of *in vivo* studies and clinical trials. However, such approaches are difficult to apply to humans since they require serious surgical intervention, multiple injections, and yet achieving poor biodistribution of the drug (93, 94). Nevertheless, such approaches are actively explored and undergoing clinical trials (95). Viral vectors, as well as methods of gene-cell therapy are also of interest for the delivery of the missing enzyme in the PNS. However, these approaches also have disadvantages, including possible genotoxicity.

For pre-symptomatic patients, bone marrow transplantation (BMT) or hematopoietic stem cell (HSC) transplantation may be helpful, but the therapeutic potential of this procedure remains controversial. It is possible that the amount of ARSA secreted by normal cells after the transplantation may not be enough to cross-correct a deficiency in the cells of the patients' nervous system (96). Thus, currently developed therapy methods for MLD require a comprehensive investigation of efficacy and safety (Tables 1, 2).

**Table 1:** Therapeutic approaches for MLD therapy undergoing *in vivo* animal studies.

**Therapeutic agent**	**Model**	**Therapeutic regimen**	**Therapy results**	**References**
**Cell therapy**				
MGTA-456 (population of CD34^+^ CD90^+^ cells)	Immunodeficient NSG mice	NA	Microglia engraftment efficiency increased by 10 times	(97)
**ERT**				
Recombinant ARSA enzyme	ARSA knockout mice	Continuous administration of the enzyme in the CSF of the right lateral ventricle of the brain for 4 weeks	Sulfatide storage in the infused hemisphere was reduced by 51–56%. Short half-life of the enzyme in the CSF (10 min)	(98)
		Intravenous administration of the 20 mg/kg enzyme for 16 weeks	Effective sulfatide removal, if the treatment begins at the pre-symptomatic stage	(87)
Chimeric ARSA protein crosslinked with the IgG domain against the human insulin receptor	WT rhesus macaque	Single intravenous administration of 55 μg/kg protein	Rapid penetration into the brain and distribution in the post-vascular parenchyma of all parts of the brain	(99)
Chimeric ARSA protein crosslinked with mouse IgG domain of transferrin receptor	ARSA knockout mice	Intraperitoneal or subcutaneous administration of 5 mg/kg protein for 5 weeks three times a week	The safety of recombinant protein was confirmed	(100)
**Gene therapy**				
AAV5-ARSA	ARSA-deficient mice with a mixed genetic background	Injection of the virus with a dose of 3 × 10^9^ vg (the sites of the injections included the cerebellar vermis, and the left and the right internal capsules)	Long-term expression of recombinant ARSA in the brain (for 3–15 months) and prevention of neuropathological and neuromotor disorders	(101)
AVV9-ARSA	Newborn ARSA knockout mice	Injection of the drug with a dose of 2 × 10^12^vg into the jugular vein of newborn mice	Long-term expression of the enzyme (up to 15 weeks) mainly in the muscles and heart, moderate expression was also found in the CNS. Sulfatide accumulation was significantly reduced in the brain and spinal cord of the treated mice	(102)
AAV1-ARSA + AAV1-FGE	ARSA knockout mice	Injection of the virus with a dose of 7.5 × 10^9^ vg into the hippocampus	Co-injection of the two vectors allowed increase in the expression and distribution of ARSA	(103)
AAVrh.10-ARSA	ARSA-deficient mice with a mixed genetic background	Intraperitoneal injection (right striatum or right ventral tegmental area)	The correction of the accumulation of certain types of sulfatides in oligodendrocytes	(104)
AAVrh.10-ARSA	WT nonhuman primates	12 injections of the vector with the dose of 1.1 × 10^11^ transducing units per the cerebral hemisphere	Enzyme activity was increased by 14–31% of normal endogenous expression and could be detected at a distance of 12–15 mm from the injection site	(93)
AAVrh.10-ARSA, AAV9-ARSA	ARSA knockout mice	Intravenous administration	AAVrh.10-ARSA more effectively infected PNS cells and reduced the sulfatide accumulation in the nervous system of MLD model mice compared to AAV9-ARSA	(105)
**Gene-cell therapy**				
HSCs genetically modified to overexpress ARSA	ARSA knockout mice	BMT	Enzyme level was increased up to 33% of normal in the CNS, up to 100% of normal in the kidneys, and up to 800% of normal in the spleen and bone marrow. The number of sulfatides was reduced, all neurophysiological disorders were normalized	(106)
Self-renewing neuroepithelial stem cells genetically modified to overexpress ARSA	Day 1 ARSA knockout mice	Transcranial injection of 100,000 cells 2 μl into the lateral ventricle with a glass capillary	Significant decrease in sulfatide accumulation in the brain	(23, 24)
Neural precursors genetically modified to overexpress ARSA	Day 1 ARSA knockout mice and day 60 ARSA knockout mice	Injection of 250,000 cells per 2 μl for postnatal day 60 mice and 200,000 cells per 2 μl in the right hemisphere	The enzyme activity reached 70% of normal expression, the accumulation of sulfatides was lower compared to the control	(107)
**Symptomatic therapy**				
Simvastatin	ARSA knockout mice	20 mg/kg/day orally for 30 days	CNS inflammation, the level of secretion of the pro-inflammatory cytokines MIP-1β and MCP-1, and brain infiltration with T-cells were decreased. 17 months after treatment, demyelination in the treated mice treated was 20% lower in the brain and 42% lower in the spinal cord compared to control animals	(108, 109)

**Table 2:** Clinical trials of the therapeutic approaches to treat MLD.

**Therapeutic agent**	**Therapeutic regimen**	**Therapy results**	**References**
HGT-1110, recombinant human ARSA enzyme	Injection of 10, 30, or 100 mg of HGT-1110 in the CSF for 38 weeks (20 injections in a week)	During therapy with a dose of 100 mg, uncertain improvements in motor function, as well as swallowing function and quality of life improvements were observed	NCT01510028
HSCs	Intravenous administration	In patients who received HSCT before the symptom onset or at a very early symptomatic stage, the disease stabilized, the rate of loss of gross motor and cognitive functions and central nervous system demyelination decreased	(110, 111)
		A study of brain tissue in two patients with MLD undergone HSCT showed that donor macrophages expressing ARSA distributed throughout the white matter, but cross-correction in resident oligodendrocytes and astrocytes did not occur, or occurred at a very low degree. However, it has been shown that HSCT can provide remyelination.	(112)
Allogeneic bone marrow-derived MSCs	Intravenous administration of 2–10 × 10^6^ MSCs/kg after BMT	In 4 out of 6 patients with MLD, an improvement in the speed of nerve conduction was observed, but no changes in the mental and physical condition of the patients were noted	(113)
	Intravenous administration of 1 × 10^6^ MSCs/kg after HSCT	In a clinical case report, stabilization of all the neurological manifestations of the disease was observed in a patient with adult MLD 40 months after the infusion	(114)
Genetically modified autologous CD34^+^ HSCs transduced with LV-ARSA	Intravenous administration of 7,2 × 10^6^ CD34^+^ HSCs/kg	Safety and efficacy have been confirmed. In all 9 patients with pre-symptomatic or with a very early symptomatic stage, the disease did not manifest or progress. However, in one patient out of 9, who already had symptoms of the disease (severe demyelination and motor and cognitive impairment) at the time of initiation of the treatment, motor activity did not improve	NCT01560182 (60)
AAVrh.10-hARSA	12 injections of AAVrh.10-hARSA with the dose of 10^12^ or 4 × 10^12^ transducing units into the white matter of the brain	In 4 children with a pre-symptomatic or very early symptomatic stage, ARSA activity in CSF, which was not detected before treatment, was significantly increased after injection, reaching 20–70% of the control values at the last assessment. In children with an early symptomatic stage, the symptoms continued to worsen, and in patients with an asymptomatic course, MLD developed, which did not differ significantly from the natural history of the disease course	NCT01801709 (115)

### Bone Marrow or Hematopoietic Stem Cell Transplantation

One of the first and most effective approaches to currently treat MLD is BMT and HSC transplantation (HSCT) (116). A number of studies have shown that BMT leads to a stable increase in the enzyme activity in leukocytes and to a halt in the development of neurocognitive and motor impairment in patients with juvenile form of MLD (110, 117). However, cohort studies have showed that in patients with juvenile MLD, demyelination continues to progress after BMT in 31% of cases. The reason of this phenomenon is unclear, but it is believed that the disease is progressing the therapy being initiated too late (117). BMT does not affect the natural course of the disease in children with late infantile and juvenile forms of MLD who receive BMT after the onset of disease symptoms (109, 117). In patients with an asymptomatic form, which are considered the most promising in terms of the effectiveness of the therapy, the disease continues to progress after BMT despite the normal level of the enzyme in blood plasma and urine that persists throughout the observation. In these patients a month after BMT, brain MRI shows abnormalities of the white matter in the frontal and occipital regions, with subsequent abnormalities in the brain progressing and epilepsy developing 2 years after BMT (108, 109).

In the case of HSCT, it has been reported that siblings with the juvenile form receiving transplantation after the onset of the first symptoms demonstrate significant decrease in psychomotor functions compared to before the onset of the disease (111). In patients with an adult MLD who received HSCT, a slowdown in the progression of the disease has also been observed (deterioration of electroencephalography (EEG) and MRI of the brain, neurological status slowed down after 12–24 months and eventually stopped, and then remained stable for 18 years after the transplantation) in comparison with non-treated patients, their older brothers or sisters, who have not received therapy. However, it should be noted that in all non-treated patients the disease manifested itself earlier compared to their siblings who received therapy, which may make the comparison with the treated patients inaccurate (118).

Based on the available retrospective cohort studies (43 patients, 27 patients) and individual clinical cases, it can be concluded that lifetime after HSCT does not associate with MLD subtype or the presence of MLD symptoms before transplantation (110, 111). In patients who received HSCT before or immediately after the symptom onset, the disease stabilizes and the rate of loss of gross motor and cognitive functions decreases. However, stabilization of disease severity observed on MRI (reduction of CNS demyelination) does not mean stabilization of peripheral nerve disease (119) and the effect of HSCT on the development of peripheral neuropathy remains controversial (110). Nevertheless, in addition to correcting ARSA deficiency, HSCT can reduce neuroinflammation in patients with MLD. It has been shown that in a patient with MLD, increased plasma levels of MCP-1, IL-1Ra, IL-8, and MIP-1β were decreased by 100 days after HSCT (17).

Recently, the results of post-mortem brain tissue examination of two patients with MLD undergone HSCT and six patients without transplantation have been reported. It has been shown that in the brain of patients with HSCT, donor macrophages expressing ARSA were distributed throughout the white matter, but no ARSA was found in resident oligodendrocytes and astrocytes. The authors suggest that either cross-correction in these cells did not occur, or ARSA could not be detected due to very low amounts. Despite this, in patients after HSCT, oligodendrocyte precursors and mature myelin-forming oligodendrocytes were present in greater numbers compared to patients without transplantation. Researchers suggest that donor macrophages can play a neuroprotective role and, thus, provide remyelination, which once again emphasizes the importance of immunomodulation (112).

Another approach similar to HSCT is cord blood cell transplantation (CBCT), since it is believed that cord blood cells more efficiently migrate to the CNS (108). Furthermore, umbilical cord blood is banked, allowing rapid selection of suitable donor and reducing the time between diagnosis and transplantation (120). Cord blood is an alternative source of HSCs, but it also contains other types of cells, such as mesenchymal stem cells (MSCs), endothelial progenitor cells and somatic stem cells (121). It has been shown that the effectiveness of CBCT does not significantly differ from the effectiveness of HSCT. Patients with early infantile and juvenile forms of MLD who receive CBCT before the symptom onset retain normal cognitive function, but suffer from severe peripheral neuropathy (122). It is also important to note that the progression of neurodegeneration in patients after CBCT is noticeably lower compared to non-treated patients (120).

A drug that can increase the percentage of microglia engraftment after CBCT to treat MLD, mucopolysaccharidosis-1H (MPS-1H), cerebral adrenoleukodystrophy (cALD), Krabbe disease and is currently under investigation. MGTA-456 is a population of CD34^+^ CD90^+^ cells that are precursors of microglia. Treatment of immunodeficient mice with MGTA-456 has resulted in increase in the efficiency of microglial engraftment by 10 times compared to control mice transplanted with CD34^+^ cord blood cells (97). The effectiveness of MGTA-456 is currently being tested in a phase II clinical trial (NCT03406962), but the results have not yet been presented.

### Mesenchymal Stem Cell Therapy

Mesenchymal stem cells (MSCs) have also been tested in an attempt to find an effective treatment for MLD. MSCs have a higher level of ARSA expression compared to monocytes from peripheral blood (123), and are also able to migrate and take root in the brain by differentiating into astrocytes (124). The majority of patients with MLD who previously received successful BMT and then transplanted with allogeneic bone marrow-derived MSCs show an improvement in the nerve conduction velocity. This suggests that either MSCs provide normal enzyme activity in or around peripheral nerves, or that they differentiate into Schwann cells *in vivo*, albeit no changes in the mental and physical condition of patients are observed (113). At the same time, Schwann cells provide a supportive growth environment for nerve cells that allows them to regenerate to some extent and to improve the nerve conduction velocity (125). A clinical case using MSCs in a 23-year-old woman with an adult form of MLD, which developed within 1 year with progressive neurological deterioration, has been described. The patient underwent allogeneic HSCT followed by infusion of allogeneic MSCs isolated from the bone marrow. Quick engraftment of MSCs without significant toxicity or side effects associated with infusion was observed. After 40 months, all the neurological manifestations of the disease were stabilized. The data obtained indicate the feasibility and potential effectiveness of allogeneic HSCT in combination with MSC infusion for patients with adult MLD (114).

### Enzyme Replacement Therapy

Enzyme replacement therapy (ERT) was one of the first approaches used to increase the level of a normal enzyme, but it encountered a number of difficulties. Experiments in MLD model mice showed that after a single dose intravenous administration of the human recombinant ARSA enzyme, most of the recombinant protein (97%) was found in the liver, 3% was found in the kidneys and <0.1% was observed in the CNS and peripheral nerves, with a half-life of ~4 days (126). The administration of a single dose of the recombinant enzyme lead to a significant decrease in the amount of sulfatides in the kidneys and peripheral tissues, but not in the brain (98). The lack of the enzyme in the brain can be explained by the fact that the BBB prevents direct transfer of the recombinant ARSA enzyme from the bloodstream to the CNS. To overcome this problem, recombinant ARSA enzyme has been injected into the CSF of MLD model mice. After 4 weeks of continuous enzyme administration in the CSF of the right lateral ventricle of the brain, a decrease in sulfatide deposits in the infused hemisphere by 51–56% was noted. However, one of the disadvantages of this method is the half-life of the enzyme in the CSF, which is only 10 min (98).

Interesting data have been obtained in the investigations of the ERT effectiveness depending on the severity of symptoms. Treatment of mice simulating the pre-symptomatic, early and progressive stages of MLD with a minimally effective dose (20 μg/kg) has shown that treatment should begin at the pre-symptomatic stage to ensure effective removal of sulfatides. No effect was seen when treatment begins after the manifestation of progressive dysfunctions of the nervous system (progressive stage of the disease). In addition, at the early stage of the disease, when the rate of nerve conduction velocity is only beginning to decrease, treatment has low efficiency, since it is impossible to achieve complete cleavage of sulfatides. This could be due to the fact that the SapB cannot penetrate into the hydrophobic nucleus of accumulated sulfatide granules and make it accessible for enzyme cleavage (87).

Another approach to increase the effectiveness of ERT was the creation of an ARSA chimeric protein crosslinked with the IgG domain against mouse transferrin receptor in mouse models (100), or with the IgG domain against human insulin receptor in experiments on rhesus macaques (99). The IgG domain allows ARSA to be delivered into the brain via the BBB using mouse transferrin or human insulin receptors, respectively. Using the rhesus macaques model, it has been shown that the chimeric protein quickly penetrates the macaque brain and distributes in the post-vascular parenchyma to all parts of the animal's brain (99). Preclinical studies in MLD model mice showed the safety of the recombinant chimeric protein (100).

Despite the promising results of ERT obtained in MLD model mice, current clinical trials show rather controversial results. For example, when 100 mg of the recombinant human ARSA enzyme (HGT-1110) was injected into the cerebrospinal fluid (CSF) for 38 weeks (????? 20 injections every other week), ambiguous improvements in motor function, as well as swallowing function and quality of life improvements have been observed (NCT01510028). In addition, a number of clinical trials of another recombinant human ARSA enzyme-based drug (HGT-1111) has not shown a slowdown in the loss of motor function and changes in the level of sulfatides in the CSF (NCT00681811, NCT00633139).

### Gene Therapy

The first steps in MLD therapy show that the use of a recombinant enzyme and transplantation of various types of stem cells can slow the progression of the disease only in asymptomatic patients and are insufficient even to stop the development of symptoms at an early stage of the disease. Therefore, the next step is the search for approaches that would help patients with progressive neuropathy.

### Adeno-Associated Viruses

The direct administration of viral vectors encoding the *ARSA* gene directly into the brain has been considered as a possible method of MLD therapy. The reason why AVVs are gaining attention in the search for an efficient method of wild-type ARSA delivery is because AAVs can transynaptically transduce neurons over a wide range from the injection site through anterograde neuronal transport (127). This ability of AAVs can allow reaching more affected parts of the brain and achieving a therapeutic effect in a minimal invasive way. However, controversial results of AAV anterograde neuronal transport should be noted. Some studies have observed anterograde neuronal transport down the axon only in specific serotypes, such as AAV5 (128) but not in AAV1, AAV2, AAV6, AAV8, or AAV9. At the same time other investigations have demonstrated AAV1, AAV2, and AAV6 anterograde transport (127, 129). Thus, conflicting results questions the AAV ability to transynaptically transduce neurons, however the therapeutic potential of AVV is being actively investigated.

The intracerebral delivery of adeno-associated virus (AAV) encoding *ARSA* can provide a fast arrest of the neurodegenerative process in the MLD patients' brain, since AAVs are one of the safest vectors for clinical use and can efficiently transduce neurons *in vivo* (130). The administration of the AAV serotype 5 (AAV5) encoding the *ARSA* gene cDNA (AAV5-ARSA) into the brain of MLD mice provides the prolonged expression of recombinant ARSA in the brain (3–15 months) and prevents neuropathological and neuromotor disorders (101).

Promising results have also been obtained using AAV9 as injection of AVV9 encoding the *ARSA* and the green fluorescent protein (*GFP*) reporter gene in the jugular vein of newborn MLD mice resulted in the prolonged expression of the enzyme (up to 15 weeks), mainly in the muscles and heart but with moderate expression found in the CNS. In the treated mice sulfatide accumulation was reduced significantly in the brain and spinal cord and not different from that in wild-type mice (102).

An approach to enhance the expression of ARSA in modified cells by injecting into MLD mouse brain AAV1-ARSA with AAV1 encoding the formylglycine-generating enzyme (FGE) gene, being necessary for the activation of sulfatases, has also been described. As a result, the level of activity and distribution of ARSA in the brain of mice were significantly increased after the co-administration of AAV1-ARSA and AAV1-FGE (103).

Among the new serotypes of the adeno-associated virus, the non-human primate AAVrh.10 spreads more efficiently from intracerebral injection sites than AAV1, AAV2, AAV5, AAV7, or AAV8 (104, 131). The injection of AAVrh.10-ARSA into the brain of 8 month old MLD mice lead to the correction of accumulation of certain types of sulfatides in oligodendrocytes, as this AAV serotype can also infect oligodendrocytes (up to 9%) (104). The safety of AAVrh.10-ARSA has also been evaluated in non-human primates (93, 132). After receiving 12 injections of the AAVrh.10-hARSA vector in an amount of 1.1 × 10^11^ transducing units per hemisphere, the enzyme activity was increased by 14–31% of normal endogenous expression and could be detected in the animals at a distance of 12–15 mm from the injection site (93). It has also been reported that AAVrh.10-ARSA more effectively infects PNS cells and reduces the accumulation of sulfatides in the nervous system of MLD model mice compared to AAV9-ARSA (105).

In a phase I/II clinical trial of this virus (NCT01801709), 4 children with a pre-symptomatic or very early symptomatic stage received 12 injections of 1 × 10^12^ or 4 × 10^12^ (depending on age) AAVrh.10-ARSA transducing units into the white matter of the brain. The activity of ARSA in CSF, which was not detected before treatment, was significantly increased after injection, reaching 20–70% of the control values at the last assessment. However, in patients with an early symptomatic stage, the symptoms continued to worsen, and in patients with an asymptomatic course, MLD developed, therefore not differing significantly from the natural course of the disease. As a result, the study was terminated due to lack of effectiveness (115).

It has also been shown that another group of AAV isolated from normal human CD34^+^ HSCs (AAVHSCs) was able to penetrate the BBB and infect neurons and glia cells in various animal models (133). Indeed, the intravenous administration of AAVHSC15-ARSA into MLD model mice increased the enzymatic activity of ARSA in key biologically significant areas of the brain, spinal cord, and PNS (134).

### Retroviruses

There have been attempts to develop therapeutic approaches based on other viruses, in particular retroviruses. Fibroblasts isolated from a patient suffering from a late infantile MLD have been genetically modified to overexpress ARSA. Transduced fibroblasts have been able to efficiently transfer ARSA to defective cells as well as to oligodendrocytes and Schwann cells through transwells *in vitro* (135). Therefore, this strategy could be a promising tool for cross-correction, but it has not been yet developed.

### Gene-Cell Therapy

In addition to virus-mediated gene therapy, a cell-mediated gene therapy approach is being actively developed where various cells are genetically modified to express wild-type ARSA (2). One of the first studies in this area was transplantation of genetically modified bone marrow cells. Before transplantation the cells were modified with a retrovirus encoding the human *ARSA* gene. The administration of genetically modified cells in 6–8 week old ARSA knockout mice with MLD resulted in an increase in enzyme level up to 33% of normal in the CNS, up to 100% of normal in the kidneys, and up to 800% of normal in the spleen and bone marrow (106). The transplantation of genetically modified cells reduced the average accumulation of lipids in the liver to about 70% of the level found in animals receiving only BMT, but no effect was observed in the kidneys and brain (106). In another study, HSCs were genetically modified with lentivirus (LV) encoding the *ARSA* gene and administrated into 6-month-old ARSA knockout mice with an MLD with severe CNS and PNS dysfunction. As a result of the therapy, the number of sulfatides were significantly decreased in mice and all neurophysiological disorders normalized (136).

The encouraging results obtained in animal models have become a platform for conducting clinical trials in patients with MLD. Current publications have described the results of the therapy in 9 of the 21 patients participating in an ongoing clinical trial (NCT01560182) (60, 137). In this trial, nine patients with a pre-symptomatic or very early symptomatic stage received 7.2 × 10^6^/kg CD34^+^ HSCs overexpressing the *ARSA* gene. Before the transplantation of genetically modified CD34^+^ HSCs, patients received chemotherapy to destroy their own hematopoietic stem cells. Two years after the transplantation, demyelinated fibers were almost not found in 5 of 7 biopsy samples, and signs of re-myelination were found in two samples. Nerve conduction velocity was both increased and decreased in different patients. In the majority of patients who received genetically modified HSC transplantation, the motor activity was similar to that of normally developing controls. However, one out of 9 patients who already had symptoms of the disease at the time of transplantation (severe demyelination and motor and cognitive dysfunction) did not improved motor activity (60).

With the discovery of the method of somatic cell reprogramming into pluripotent cells, it became possible to use the patient's own cells as a source of autologous cells for transplantation. IPSCs obtained from skin fibroblasts of an MLD patient have been reprogrammed into self-renewing neuroepithelial stem cells, which were subsequently genetically modified to overexpress ARSA. The obtained cells were transplanted into the telencephalon of 1-day-old ARSA^−/−^ mice, which led to a significant decrease in the accumulation of sulfatides in the mouse brain (24). In a similar study, neural precursors overexpressing ARSA were also obtained from iPSCs (107). However, in this study the cells were transplanted not only to 1-day-old mice, but also to 60-day-old mice with MLD. It was shown that precursors differentiated into astrocytes and oligodendrocytes, while in the first study (24) no oligodendrocyte markers were found. In the transplanted mice, the enzyme activity reached 70% of normal and the accumulation of sulfatides was lower compared to the control (107).

In general, gene-cell MLD therapy seems to be a rather promising approach because, as cells are able to “transfer” the enzyme to the mutant nervous system cells of the patients having overcome the BBB. Such cross-correction mechanism is based on the fact that a small part of the newly synthesized soluble enzyme is released from the cell in the intercellular space instead of entering the lysosome. Outside the cell the enzyme can enter the neighboring cell by endocytosis and be delivered into the lysosome via M6P receptors (24). Thus, it may seem that significant overexpression of the enzyme in genetically modified cells can ensure the spread of the enzyme over long distances along the nervous system and provide a significant therapeutic effect. However, recent articles on another LSD showed that this might not have a big impact and in case of hematopoietic stem cell transplantation as the therapeutic effect of cells expressing the normal enzyme can be mediated by phagocytic response of healthy macrophages, rather than cross-correction (138).

### Symptomatic Therapy

Since there is no sufficiently effective therapy for MLD, patients have to deal with the need to alleviate the symptoms of the developing disease. Symptomatic therapy includes many different approaches aimed to relieve many clinical symptoms, however the effectiveness of many drugs used is not confirmed (139).

As a symptomatic treatment for spasticity, intrathecal administration of baclofen is used. Direct administration of baclofen to the CNS is preferable, since when taken orally, a smaller part of the drug overcomes the BBB. Therefore, a lower dose can be used compared to oral administration when administered intrathecally so that many side effects can be avoided. The initial dose of baclofen during intrathecal administration may begin with a dose of 40–100 μg per day and be adjusted (often increased) due to the progressive nature of the disease (140, 141).

Warfarin may also be prescribed as a drug for the symptomatic treatment of MLD. Warfarin is an antagonist of vitamin K, which regulates the biosynthesis of sphingolipids. It is known that warfarin can decrease the level of sulfatides in the brain of rats and mice. However, in a study of 13 MLD patients who could not have BMT and who have been receiving warfarin for 45 days, no beneficial effects of warfarin were shown (142, 143).

Demyelination of the nervous system cells during the MLD progression has also been associated with neuroinflammation (109). Therefore, anti-inflammatory therapy may be effective in preventing neuroinflammation and reducing the rate of demyelination in MLD patients. To prove this, an experiment where MLD model mice received orally 20 mg/kg/day of simvastatin for 30 days was performed. Simvastatin was chosen since it overcomes the BBB better than other statins and inhibits the MAPK signaling pathway through which signals of the pro-inflammatory cytokines MIP-1α, MIP-1β and MCP-1 are transmitted (144). The administration of the drug lead to a decrease in the CNS inflammation, a decrease in the level of secretion of the pro-inflammatory cytokines MIP-1β and MCP-1, and a decrease in the brain infiltration with T-cells., Demyelination in the treated with simvastatin mice was 20% lower in the brain and 42% lower in the spinal cord compared to control animals 17 months after the therapy (109). These encouraging data now require validation in clinical trials.

Another therapeutic approach to prevent neuroinflammation is immunotherapy with intravenous immunoglobulins (IVIg), which is currently widely used to treat autoimmune diseases of the PNS. IVIg are an immunologically active protein fraction isolated from human donor plasma or serum. The active component of the drug is the IgG, which has the antibody-like activity against various viruses and bacteria, as well as non-specific and immunoregulatory activity, which manifests itself in an increase in the body's resistance to infectious diseases and anti-inflammatory effect. IVIg are able to modulate complement activation, suppress idiotypic antibodies, saturate Fc receptors on macrophages and suppress various inflammatory mediators, including cytokines, chemokines, and metalloproteinases (145). Thus, IVIg have an immunosuppressive effect, inhibiting inflammatory reactions and the activation of microglia, which prevents further damage of neurons associated with inflammation.

It has been shown that the effects of IVIg therapy can be mediated by an increase in the level of macrophage colony-stimulating factor (M-CSF) and MCP-1, which has an immunomodulating effect. Individual case study reports of the IVIg use for the MLD treatment are described. However, most reports have shown no significant improvement after IVIg-based treatment (146–148). For example, IVIg infusions (loading dose of 2 g/kg, maintenance dose of 0.4 g/kg every 2 weeks) for a MLD patient with severe peripheral neuropathy did not lead to an improvement in clinical symptoms, moreover, an increase in symptoms was detected, after which IVIg infusions were canceled (148).

However, it is believed that immunomodulation may temporarily be useful for the treatment of neuropathy in the early stages of MLD. Functional improvement was observed in a 4-year-old girl with MLD during immunomodulating therapy with IVIg, but cognitive and motor impairment were shortly (149). However, another study has shown that after two courses of IVIg (one course was for 5 days, a dose of 2 g/kg/day) in a patient with MLD, respiratory failure and ptosis were completely eliminated (150). Thus, the benefits of IVIg in the treatment of MLD cannot be unambiguously defined and more extensive studies are required to determine the effectiveness and validity of immunoglobulin therapy in MLD.

Prednisolone is also prescribed for the treatment of neuroinflammation (13). It has been suggested that prednisolone may provide short-term functional improvement in patients with MLD (146). However, these data are not in line with another study, where after oral administration of prednisolone (the dose started from 100 mg/day and was gradually decreased to 20 mg/day for 4 weeks) no changes in neurological, neuropsychological signs, EEG and MRI results were observed after 6 months (58). More often, prednisolone is prescribed for patients with MLD for other purposes, such as the prevention and treatment of graft vs. host disease after BMT (151–153).

## Conclusions

Unlike other LSDs, there is currently no approved specific therapy for MLD. Allogeneic HSC transplantation helps some patients to delay the disease onset, but is not able to completely stop the progression. In addition, it carries the risk of complications, including fatal ones. One of the most dangerous adverse effects is graft vs. host disease (GVHD), in which donor immune system cells begin to attack recipient tissue due to the incompatibility of human leukocyte antigens (HLA) proteins. It can be assumed, that PBCT or MSC transplantation may become a potentially safer and more effective method of therapy, but so far, the observation period and the number of treated patients are insufficient to draw final conclusions. The method of ERT is being developed, such as the intrathecal injection of human recombinant ARSA enzyme. Intrathecal administration circumvents the problem of passing the BBB. However, according to the latest published data, even intrathecal ERT could only slightly slow down the disease progression. At the same time, ERT has shown effectiveness in *in vivo* studies in MLD mice, which once again underlines the difficulties in translating animal research into clinical benefit.

Thus, most patients receive only symptomatic therapy such as antiepileptic drugs in case of seizures, muscle relaxants or physiotherapy, as well as anti-inflammatory therapy with prednisolone or IVIg. Although this relieves their condition, it does not affect the cause and pathogenesis of the disease, hence not affecting the speed of the disease progression.

Gene and combined gene-cell therapy are considered new promising methods for MLD therapy. In this field the use of recombinant AAVrh.10-hARSA and genetically modified autologous CD34^+^ HSCs transduced with LV-ARSA are worth mentioning. According to the latest available published data posted by the company Orchard Therapeutics commercializes this technology, the observation period for the first patient who received autologous CD34^+^ HSCs transduced with LV-ARSA has already exceeded 8 years, and he has been developing in the same way as his healthy peers. In the first half of 2020, Orchard Therapeutics plans to register the method of MLD therapy at the European Medical Agency (https://ir.orchard-tx.com/index.php/news-events/presentations).

Thus, gene and gene-cell therapy have so far shown safety and efficacy in clinical trials. This evidence opens new possibilities for the treatment of pre-symptomatic or early symptomatic stage MLD patients. Further studies of already proven methods may also provide opportunities for the treatment of patients with terminal symptoms of the disease.

## Author Contributions

VS, AS, DC, and AR: conceptualization. AS, DC, VS, and AM: writing-original draft preparation. CA, VS, and AR: writing-review and editing. KK: visualization. VS and AR: supervision. All authors have read and agreed to the published version of the manuscript.

## Conflict of Interest

The authors declare that the research was conducted in the absence of any commercial or financial relationships that could be construed as a potential conflict of interest.
